# Holistic Human Caring Perception and Perceived Professional Benefit Among Nurses in China: A Multihospital Cross‐Sectional Study

**DOI:** 10.1155/jonm/3967540

**Published:** 2026-07-11

**Authors:** Jiayi Chen, Jiachun Lu, Ying Liu, Xiaohan Hu, Yingai Cui, Peng Xiong, Lingjuan He, Yanli Hu

**Affiliations:** ^1^ School of Nursing, Guangzhou Medical University, Guangzhou, China, gzhmc.edu.cn; ^2^ Department of Public Health and Preventive Medicine, School of Medicine, Jinan University, Guangzhou, China, jnu.edu.cn; ^3^ Department of Nursing Research, Guangdong Medical University, Dongguan, China, gdmu.edu.cn; ^4^ Department of Medical Oncology, Huizhou Central People’s Hospital, Huizhou, China

**Keywords:** holistic human caring perception, nurses, perceived professional benefit

## Abstract

**Aim:**

This study aimed to examine nurses’ holistic human caring perception and its relationship with perceived professional benefit.

**Background:**

Holistic human caring perception is crucial for holistic nursing and nursing practice. Comprehending its link with nurses’ perceived professional benefit benefits nursing administrators and policymakers. However, this relationship among nurses is still understudied.

**Methods:**

A cross‐sectional study was conducted, involving 276 nurses recruited from 23 hospitals across China. Data were collected through an online questionnaire, which assessed nurses’ sociodemographic characteristics, holistic human caring perception, and perceived professional benefit. Statistical analyses included Pearson’s correlation analysis, *t*‐tests, analysis of variance (ANOVA), and multiple linear regression analysis to examine the relationships and influencing factors.

**Results:**

The mean score of holistic human caring perception among nurses was 153.10 ± 27.36, indicating a moderate level. A significant positive correlation was found between holistic human caring perception and perceived professional benefit (*p* < 0.001). The multiple linear regression model (*n* = 276) explained 58.2% of the variance (adjusted *R* ^2^ = 0.582) and identified holistic human caring perception, employment type, nurse position, and exercise habits as the primary factors associated with nurses’ perceived professional benefit (*p* <  0.05).

**Conclusion:**

The findings suggest that enhancing nurses’ holistic human caring perception is positively associated with their perceived professional benefit. Therefore, targeted interventions aimed at improving holistic human caring perception may serve as an effective strategy to promote nurses’ professional satisfaction and overall well‐being.

**Implication for Nursing Managers:**

The results offer practical guidance for nursing managers to design and implement interventions that strengthen nurses’ humanistic care capabilities, ultimately improving both nurse satisfaction and the quality of patient care.

## 1. Background

Nurses comprise nearly 69% of the global healthcare workforce [1]. They carry essential responsibilities in high‐stakes clinical settings. They often face emotionally distressing events, including patient suffering and death. These experiences place them at high risk for psychological trauma and occupational burnout [2]. Additionally, nurses’ physical and psychological well‐being is under considerable strain due to factors such as prolonged stress, high attrition, and an aging workforce [3–5]. As a result, the profession has become less attractive worldwide, and nursing shortages continue to worsen [6, 7]. Burnout and high turnover intention remain major concerns [8, 9]. These issues highlight the need for a deeper understanding of factors that support nurses’ well‐being. Research grounded in positive psychology suggests that stronger psychological resources can improve job satisfaction, strengthen professional identity, reduce burnout, and lower attrition [10, 11]. This focus on well‐being also connects to broader discussions about the moral and relational foundations of caring within healthcare organizations.

The foundations of organizational care can be traced back to Christian ideals of compassion and altruism, originally perceived as divinely inspired. Over time, however, the notion of charity has undergone transformation under humanistic and postmodern lenses, increasingly emphasizing the individual’s role as the source of moral action [12]. Holistic human caring emphasizes mutual support, empathy, resource sharing, and collaborative relationships among individuals [13]. Watson (1988) suggested that human beings possess intrinsic energy and potential and that acts of caring can amplify this inner strength by cultivating a broader energetic connection [14]. The centrality of care in human‐focused disciplines has gained widespread recognition. Dr. Jean Watson’s influential theory, introduced in 1979, identified holistic human caring as the essential and foundational element of nursing [15]. She argued that effective care involves not only clinical skills but also emotional and psychological support. Without the presence of care, nursing loses its meaning, making care the cornerstone of both quality practice and professional advancement [16]. As essential providers of care, nurses hold a crucial role in delivering this humanistic dimension. The care delivered in nurturing and supportive environments is often more profound than that found in other contexts [17]. Therefore, gaining insights into nurses’ experiences within their professional roles is key to promoting both their well‐being and that of their patients.

Perceived professional benefit (PPB) denotes an individual’s subjective recognition of the rewards and gains from their occupation. This concept reflects a positive emotional state, acknowledging that one’s profession fosters holistic personal development [18, 19]. In the nursing context, PPB refers to the value and growth nurses associate with their occupational experiences, encompassing both personal and career development opportunities [20]. According to the Broaden‐and‐Build Theory, positive emotional states enhance individuals’ cognitive and behavioral capacities, laying the groundwork for their overall development [21]. Previous studies suggest that the factors influencing PPB can be understood at three levels. At the individual level, PPB is positively associated with psychological resilience and well‐being [19, 22, 23]. Personality also plays a role. Neuroticism is negatively related to PPB, whereas conscientiousness, agreeableness, openness, and extraversion show positive associations [24]. At the interpersonal level, supportive relationships contribute to higher PPB. Peer support, supervisor support, and mentorship are linked to better psychological outcomes and stronger professional meaning [25]. Studies also show that supervisor support and perceived organizational support predict nurses’ retention intentions and professional well‐being [26]. At the organizational level, mental workload and high‐pressure environments reduce PPB and increase emotional exhaustion and disengagement [4, 24, 27]. Positive work environments, higher spiritual care competency, and supportive leadership are associated with higher PPB [19, 22]. PPB may also reduce transition shock, lower burnout and turnover intention, and improve nursing quality [23].

Humanistic caring plays an important role in nurses’ professional growth, and spiritual care is a central component of this caring orientation [28]. Research demonstrates that nurses who effectively manage occupational stress and recognize the intrinsic value of their profession tend to show enhanced empathy and a stronger understanding of nursing as a caring science [29, 30]. However, despite the growing attention to PPB, little is known about how nurses’ perceptions of holistic human caring contribute to their sense of professional gain. Although prior studies recognize that nurses work within complex social systems—including self‐care practices [31], work–family balance [32], and broader social and supervisory support [33]—these factors have mostly been examined independently and without integration into frameworks of PPB. Existing research seldom incorporates caring perceptions into PPB models, and few studies use multihospital samples that reflect broader contextual influences. This gap limits understanding of how caring‐related perceptions shape key professional outcomes, including job fulfillment, professional identity, and long‐term career development.

In light of these concerns, this study sought to examine the relationship between Chinese nurses’ perceptions of holistic human caring and their PPB, with the goal of informing strategies to cultivate a more resilient and sustainable nursing workforce.

## 2. Methods

### 2.1. Study Design and Participants

This cross‐sectional study was carried out between January and February 2025, involving the participation of 276 licensed clinical nurses from 23 general hospitals across six provinces in China. These hospitals represented different hospital levels within China’s national grading system, including Level I, Level II, and Level III general hospitals. All hospitals offered a full range of medical and nursing services.

This cross‐sectional study was reported in accordance with the STROBE guidelines (see Appendix 1: Supporting Information).

A convenience sampling approach was used. A total of 318 questionnaires were distributed, and 276 valid responses were included in the final analysis (response rate: 86.8%). Questionnaires were excluded if (1) more than 80% of the items were answered with the same option, suggesting inattentive responding, or (2) the total completion time was less than 5 min, indicating insufficient engagement with the survey content. Eligibility criteria for inclusion required that participants (1) held active registration as clinical nurses and (2) had completed standardized orientation or onboarding training. Individuals were excluded if they (1) were temporarily visiting professionals from other institutions or (2) were not present at work during the data collection period.

The minimum required sample size was calculated using G∗Power 3.1 for multiple linear regression. A medium effect size (*f* ^2^ = 0.15), a power of 0.80, and an alpha level of 0.05 were assumed. With 27 predictors, the recommended minimum sample size was 196. The final sample of 276 participants exceeded this requirement, providing sufficient power for the planned analyses.

### 2.2. Procedure for Data Collection

Following administrative approval from each hospital involved, one or two designated research personnel were assigned to oversee recruitment and data collection in each facility. Invitations were distributed via digital messaging tools such as WeChat, informing potential participants of the research purpose, estimated completion time (approximately 10–20 min), and inclusion/exclusion criteria. These details were also restated in the introductory section of the questionnaire. After participants provided electronic informed consent, data were gathered through the professional online survey platform “SO JUMP.”

### 2.3. Instruments

Data were collected using a structured, self‐administered online questionnaire comprising three core parts: demographic information, the Holistic Human Caring Perception Scale, and the Perceived Professional Benefit Scale.

A structured sociodemographic questionnaire was used to collect information on nurses’ personal and work‐related characteristics. The selection of variables followed previous empirical studies and theoretical evidence demonstrating that demographic and occupational factors may influence nurses’ caring perceptions, professional attitudes, and work‐related outcomes, such as job satisfaction, burnout, and intention to leave [34, 35]. To describe the sample and control potential confounders, the questionnaire assessed hospital level, age, educational background, working years, marital status, number of children, night‐shift frequency, employment type, professional title, nurse position, place of birth, only‐child status, religious belief, exercise habits, and monthly income. All variables and response options were standardized across study sites.

The Holistic Human Caring Perception Scale, originally developed by Chen (2023) [36], was used to assess nurses’ perceptions in this domain. It includes 40 items divided into six categories: interactions with department leadership and colleague support (14 items), family support (7 items), self‐care (7 items), peer and friend support (5 items), hospital leadership support (4 items), and patient–family support (3 items). Responses were rated on a five‐point Likert scale, yielding total scores ranging from 40 to 200. The scale demonstrated excellent internal consistency in this study, with a Cronbach’s alpha coefficient of 0.98.

PPB was evaluated using the instrument developed by Hu (2020) [31]. This tool contains 17 items grouped into five thematic dimensions: positive occupational perception (3 items), good nurse–patient relationship (4 items), recognition from family, relatives, and friends (3 items), sense of belonging to the team (3 items), and self‐growth (4 items). Each item was rated on a five‐point Likert scale, producing a cumulative score between 17 and 85. Reliability was confirmed with Cronbach’s alpha values ranging from 0.74 to 0.85 in this sample.

### 2.4. Ethical Considerations

The research protocol received approval from the Ethics Committee of Guangzhou Medical University (Approval No. L202306002). Prior to enrollment, all participants were provided with a full explanation of the study’s goals, procedures, and their rights, including the right to withdraw at any time. Informed consent was obtained digitally. Participants were assured that their responses would remain confidential and anonymous, and they completed the survey independently via the online platform.

### 2.5. Statistical Analysis

Data analysis was performed using IBM SPSS Statistics Version 25.0 (IBM Corp., Armonk, NY, USA). To assess normality, quantile–quantile plots were reviewed for both holistic caring perception and PPB scores. Descriptive statistics, including means, standard deviations, and percentages, were used to summarize participant characteristics and variable distributions. Group comparisons across sociodemographic factors were conducted using independent‐samples *t*‐tests and one‐way analysis of variance (ANOVA). Relationships between holistic caring perception and PPB were examined using Pearson correlation coefficients. In addition, stepwise multiple linear regression analysis was employed to determine the key variables influencing PPB among nurses.

## 3. Results

### 3.1. Demographics

Among the 276 participating nurses, over half of the respondents (51.8%) fell within the age range of 20–30 years. In terms of educational qualifications, 66.3% held a bachelor’s degree. Most participants (88.8%) were employed in level III hospitals. Additionally, 85.9% had 0‐10 years of clinical nursing experience, with 59.8% reporting an average of four to seven night shifts each month.

Regarding personal characteristics, 68.5% of participants were married, and 38.4% had no children. A small proportion (6.9%) were only children, while the majority (70.7%) hailed from rural areas. Economically, the largest group (55.8%) reported a monthly income of ≥8000 CNY. Remarkably, only 2.2% of participants engaged in regular physical exercise.

Statistical analyses, including *t*‐tests and ANOVA, revealed significant differences in nurses’ PPB across several demographic variables, including age, educational background, working years, professional title, nurse position, exercise habits, and monthly income (including bonuses) (*p* < 0.05) (see Table 1).

**TABLE 1:** Comparison of nurses’ perceived professional benefits among nurses with different sociodemographic characteristics (*N* = 276).

Sociodemographic characteristics	N (%)	Professional benefits (total±SD)	t/F	*p*
Hospital level				
Level I	7 (2.5)	64.57 ± 15.75		
Level II	24 (8.7)	66.38 ± 9.67	1.972	0.141
Level III	245 (88.8)	70.36 ± 11.87		
Age				
20–30	143 (51.8)	68.35 ± 11.96		
31–40	105 (38.0)	70.17 ± 12.10	5.795	0.003
> 40	28 (10.1)	76.50 ± 7.43		
Educational background				
Secondary vocational school	5 (1.8)	62.60 ± 18.70		
Associate degree	88 (31.9)	67.83 ± 10.97	3.206	0.042
Bachelor’s degree	183 (66.3)	71.05 ± 11.92		
Working years				
0–10	237 (85.9)	69.05 ± 12.14		
11–20	21 (7.6)	74.29 ± 9.08	4.202	0.016
> 21	18 (6.5)	75.56 ± 7.55		
Marital status				
Single	83 (30.1)	68.34 ± 11.66	1.007	0.367
Married	189 (68.5)	70.51 ± 11.98		
Divorced	4 (1.4)	71.50 ± 7.42		
Number of children				
0	106 (38.4)	68.95 ± 11.58		
1	74 (26.8)	69.45 ± 12.98	0.978	0.377
≥ 2	96 (34.8)	71.20 ± 11.20		
Night shift per month				
0–3	104 (37.7)	71.03 ± 12.37		
4–7	165 (59.8)	69.21 ± 11.46	0.819	0.442
≥ 8	7 (2.5)	68.29 ± 13.31		
Employment type				
Contract‐based staff	181 (65.6)	70.08 ± 12.30		
Agency‐hired staff	28 (10.1)	69.86 ± 10.22	0.107	0.899
Permanent staff	67 (24.3)	69.30 ± 11.34		
Professional title				
Nurse	56 (20.3)	68.48 ± 10.55		
Senior nurse	114 (41.3)	68.66 ± 13.11		
Lead nurse	84 (30.4)	70.65 ± 11.31	2.642	0.034
Associate chief nurse	16 (5.8)	75.38 ± 6.73		
Chief nurse	6 (2.2)	80.17 ± 7.76		
Nurse position				
Staff nurse	245 (88.8)	69.11 ± 12.02	−3.039	0.003
Nurse manager	31 (11.2)	75.87 ± 8.28		
Place of birth				
Urban	60 (21.7)	70.55 ± 11.73	0.372	0.689
Rural	195 (70.7)	69.87 ± 11.68		
Urban–rural fringe	21 (7.6)	67.95 ± 11.04		
Only child				
Yes	19 (6.9)	70.32 ± 11.09	0.170	0.865
No	257 (93.1)	69.84 ± 11.92		
Religion				
Buddhism	22 (8.0)	73.59 ± 9.41	2.304	0.102
Other religions	1 (0.4)	52.00		
None	253 (91.7)	69.62 ± 11.96		
Exercise habits				
Never	23 (8.3)	69.48 ± 14.54	3.515	0.008
Seldom	130 (47.1)	67.58 ± 11.65		
Sometimes	88 (31.9)	72.64 ± 11.70		
Often	29 (10.5)	72.28 ± 8.86		
Always	6 (2.2)	76.33 ± 8.85		
Monthly income (including bonuses)				
2000–3999 CNY	26 (9.4)	66.58 ± 12.02	2.932	0.034
4000–5999 CNY	36 (13.0)	70.94 ± 10.74		
6000–7999 CNY	60 (21.7)	66.85 ± 13.02		
≥ 8000 CNY	154 (55.8)	71.35 ± 11.35		

### 3.2. Holistic Human Caring Perception (HHCP)

Quantile–quantile plot analysis confirmed that the distributions of HHCP and PPB approximated normality. Therefore, descriptive statistics—means and standard deviations—were used to summarize these variables.

The overall mean score for HHCP was 153.10 ± 27.36 (out of 200). Subscale averages were as follows: department leadership and colleague support (54.04 ± 11.08, out of 70), family support (27.76 ± 5.76, out of 35), self‐care (26.76 ± 4.87, out of 35), peer and friendship support (19.84 ± 4.05, out of 25), hospital leadership support (13.27 ± 3.84, out of 20), and patient–family support (11.43 ± 2.40), out of 15). Collectively, these findings suggest that participants reported a moderate level of HHCP (see Table 2).

**TABLE 2:** Descriptive analyses of holistic human caring perception among nurses and nurses’ perceived professional benefits (*N* = 276).

Variables	Min.	Max.	Mean	SD
Holistic human caring perception				
Department leadership and colleague support	16.00	70.00	54.04	11.08
Family support	7.00	35.00	27.76	5.76
Self‐care	14.00	35.00	26.76	4.87
Peer and friend support	8.00	25.00	19.84	4.05
Hospital leadership support	5.00	20.00	13.27	3.84
Patient–family support	5.00	15.00	11.43	2.40
Total score	74.00	200.00	153.10	27.36
Nurses’ perceived professional benefits				
Positive occupational perception	3.00	15.00	11.50	2.80
Good nurse–patient relationship	7.00	20.00	16.96	2.83
Recognition from family, relatives, and friends	3.00	15.00	12.31	2.47
Sense of belonging to the team	6.00	15.00	12.39	2.14
Self‐growth	8.00	20.00	16.70	2.86
Total score	34.00	85.00	69.87	11.84

### 3.3. PPB

The mean overall score for PPB among the participating nurses was 69.87 ± 11.84 (out of 85). Subscale averages were as follows: positive occupational perception (11.50 ± 2.80, out of 15), good nurse–patient relationship (16.96 ± 2.83, out of 20), recognition from family, relatives, and friends (12.31 ± 2.47, out of 15), sense of belonging to the team (12.39 ± 2.14, out of 15), and self‐growth (16.70 ± 2.86, out of 20). Overall, these findings indicate that clinical nurses perceived their professional roles as moderately to highly rewarding (see Table 2).

### 3.4. Relationship Between HHCP and PPB

A significant positive correlation was observed between HHCP and PPB. As presented in Table 3, all calculated correlation coefficients were statistically significant (*p* < 0.01) and ranged from 0.431 to 0.750. As detailed in Table 3, the total scores for both constructs were strongly correlated (*r* = 0.744). The strength of these associations was particularly notable, with the several correlations falling within a strong range (e.g., > 0.60). For instance, “Departmental leadership and colleague support” showed a very strong correlation with “Sense of belonging to team” (*r* = 0.710). These results demonstrate that higher levels of HHCP are strongly linked to greater PPB among nurses.

**TABLE 3:** Correlation analyses (R values) between holistic human caring perception and perceived professional benefits (*N* = 276).

	Positive occupational perception	Good nurse–patient relationship	Recognition from family, relatives, and friends	Sense of belonging to the team	Self‐growth	Total score for perceived professional benefits
Departmental leadership and colleague support	0.590^∗∗^	0.614^∗∗^	0.505^∗∗^	0.710^∗∗^	0.691^∗∗^	0.687^∗∗^
Family support	0.499^∗∗^	0.573^∗∗^	0.472^∗∗^	0.617^∗∗^	0.591^∗∗^	0.608^∗∗^
Self‐care	0.503^∗∗^	0.507^∗∗^	0.500^∗∗^	0.577^∗∗^	0.577^∗∗^	0.588^∗∗^
Peer and friend support	0.540^∗∗^	0.607^∗∗^	0.560^∗∗^	0.649^∗∗^	0.631^∗∗^	0.660^∗∗^
Hospital leadership support	0.539^∗∗^	0.468^∗∗^	0.431^∗∗^	0.551^∗∗^	0.531^∗∗^	0.557^∗∗^
Patient–family support	0.620^∗∗^	0.557^∗∗^	0.499^∗∗^	0.643^∗∗^	0.644^∗∗^	0.656^∗∗^
Total score for holistic human caring perception	0.644^∗∗^	0.664^∗∗^	0.580^∗∗^	0.750^∗∗^	0.732^∗∗^	0.744^∗∗^

^∗∗^ *p* < 0.01.

### 3.5. Factors Associated With PPB

HHCP and all demographic characteristics were entered into the multiple linear regression model. The findings showed that HHCP was the strongest and most significant positive predictor of PPB. In addition, nurse position and exercise habits were positively associated with the outcome. Employment type was also a significant predictor of PPB. Nurses in contract‐based positions reported higher PPB than those in permanent posts. The final model accounted for 58.2% of the variance in PPB (adjusted *R* ^2^ = 0.582; see Table 4).

**TABLE 4:** The multiple linear regression analysis of perceived professional benefits among nurses (*N* = 276).

Variables	B	SE	Beta	*t*	*p*	VIF
Holistic human caring perception	0.311	0.018	0.719	17.701	< 0.001	1.088
Hospital level	0.380	1.244	0.013	0.306	0.760	1.236
Age	1.658	1.291	0.094	1.284	0.200	3.504
Educational background	2.185	1.187	0.095	1.841	0.067	1.761
Working years	0.917	0.841	0.060	1.090	0.277	1.965
Marital status	1.585	1.444	0.065	1.098	0.273	2.291
Number of children	−0.158	0.894	−0.011	−0.177	0.860	2.751
Night shift per month	1.734	1.016	0.077	1.706	0.089	1.356
Employment type	−1.577	0.686	−0.114	−2.298	0.022	1.614
Professional title	−0.451	0.916	−0.035	−0.492	0.623	3.381
Nurse position	3.710	1.873	0.099	1.981	0.049	1.696
Place of birth	−0.768	0.920	−0.034	−0.835	0.404	1.091
Only child	1.992	1.889	0.043	1.005	0.292	1.081
Religion	1.113	0.770	0.058	1.445	0.150	1.056
Exercise habits	1.154	0.566	0.085	2.039	0.042	1.173
Monthly income	−0.218	0.609	−0.018	−0.357	0.721	1.769

*Note:* *R* ^2^ = 0.607; adjusted *R* ^2^ = 0.582; *F* = 24.979; *p* < .0001.

## 4. Discussion

### 4.1. PPB

The mean score for PPB among nurses in this study was 69.87 ± 11.84 (out of 85), which shows some differences when compared to findings from previous studies. Earlier research has generally indicated that Chinese nurses tend to report moderate levels of PPB [37]. In this study, all five subdimension scores exceeded 76.5% of their respective maximum values. The highest mean score was observed for nurse–patient interactions, while the lowest was observed for positive perceptions of the nursing profession.

While nurses reported moderate to high levels of PPB, several dimensions showed relatively lower scores, including positive occupational perception, recognition from family and friends, sense of belonging to the team, and self‐growth. These results suggest that enhancing nurses’ occupational perception requires not only clinical skill development but also broader social recognition and support from colleagues, relatives, and the wider community. A supportive external environment is essential for shaping nurses’ professional outlook and strengthening their commitment to the profession.

Moreover, improving the management of nursing teams and fostering a positive organizational culture can contribute significantly to the professional growth of nurses. Institutions that focus on career development and create environments that support personal and professional advancement can play a pivotal role in ensuring the sustainable growth of the nursing workforce.

### 4.2. Factors Associated With Nurses’ PPB

Several factors showed significant associations with nurses’ PPB in this study. Employment type was significantly associated with PPB. Interestingly, our study found that contract‐based nurses reported higher PPB than those in permanent positions. This employment pattern can also be interpreted through the dimension of positive occupational perception within PPB. Contract‐based nurses, who are often younger and highly motivated, tend to experience a strong sense of achievement and gratitude when working in a tertiary hospital, especially given the competitive recruitment environment in China. These feelings enhance their positive evaluation of their professional role and reinforce their sense of value, growth, and recognition. In contrast, nurses in permanent positions may hold higher expectations for career development due to their educational background or earlier entry into the system. When promotion opportunities are limited or long‐term night‐shift duties accumulate, the gap between expectations and actual experiences may weaken their positive occupational perception and reduce their PPB. Younger contract‐based nurses also adapt more easily to smart technologies and digital workflows, which strengthens their sense of competence and further enhances their positive occupational perception. This finding also reflects a broader structural issue within the nursing workforce—the lack of standardized guidelines for professional ethics and role expectations. Strengthening relevant regulations and ensuring their consistent implementation are essential for promoting equitable career development and fostering a positive professional identity among nurses across different employment types [38].

Nurse position was also positively associated with PPB. Nurse managers often have greater autonomy and a wider scope of decision‐making, which can enhance their sense of professional competence and contribution. These roles also involve more frequent team coordination and communication, helping nurses build stronger connections with their colleagues and reinforcing their sense of belonging within the team. In addition, the responsibilities of guiding junior staff and supporting clinical decision‐making can strengthen nurses’ awareness of their professional value, while increased patient and family recognition further contributes to a more positive perception of their work [39]. These advantages can strengthen their professional identity and sense of contribution.

Exercise habits were another factor associated with PPB. Nurses who engage in regular physical activity may improve their physical capacity and energy, allowing them to handle routine tasks more comfortably and demonstrate greater resilience during unexpected clinical situations. Better physical health and emotional regulation also enable nurses to respond to patients with greater patience and stability, which supports more positive nurse–patient interactions. These experiences may help nurses recognize the value and meaning of their professional role, strengthening their positive view of the profession and contributing to higher levels of PPB.

### 4.3. Perception of Holistic Human Caring

The findings from this study indicate that nurses demonstrated a moderate level of HHCP, with an average score of 153.10 ± 27.36 (out of 200). Prior studies have shown that Chinese nurses vary in their capacity for humanistic care, with some reporting lower scores compared to their peers in Western nations [40]. One likely reason for this gap is the relatively limited integration of humanistic care concepts in nursing curricula, as well as the scarcity of structured training programs focused on this aspect of care in Chinese clinical settings [41]. Consequently, nurses working in public hospitals may encounter obstacles in developing and applying holistic caring approaches.

Moreover, the growing elderly population has led to an escalating demand for healthcare services, placing additional strain on existing resources. Within such a resource‐limited and time‐constrained environment, nurses may be compelled to focus more on biomedical tasks and routine procedures, thereby reducing the time and energy they can devote to meaningful patient engagement [42]. This shift in priorities may inadvertently hinder the delivery of comprehensive, patient‐centered care.

### 4.4. Association Between Nurses’ HHCP and PPB

This study found a strong positive association between nurses’ HHCP and their PPB. HHCP was the strongest predictor in the regression model, indicating that nurses who hold a deeper awareness of holistic human caring experience greater meaning, affirmation, and value in their work.

Nurses who perceive themselves as both providing and receiving care are likely to experience affirmation and support, which strengthens their professional identity. According to Watson’s Human Caring Theory [14], such caring interactions help nurses find meaning in their work and foster a sense of connection with others, thereby deepening their commitment to caring practice. A stronger professional identity subsequently promotes greater work engagement. As nurses become more engaged, they are more inclined to invest effort in expanding their cognitive capacities and enhancing their practical skills [23, 29]. This gradual accumulation of positive experiences aligns with the Broaden‐and‐Build Theory, which posits that positive emotional states broaden individuals’ thought–action repertoires and support the development of lasting personal resources [43]. In the nursing context, enhanced cognitive and behavioral resources create more opportunities for recognition, accomplishment, and career‐related rewards, ultimately strengthening nurses’ PPB [28].

The nurse–patient relationship also plays a central role in shaping nurses’ professional perceptions [44]. Positive nurse–patient interactions enhance emotional connection, job satisfaction, and caring confidence, thereby strengthening PPB [45]. Patient appreciation and trust can validate nurses’ efforts and reinforce their sense of value within the healthcare team, ultimately increasing professional fulfillment.

Nurses with higher HHCP are more likely to pick up on subtle nonverbal cues, such as discomfort or anxiety, enabling timely interventions that improve the patient experience. Watson emphasizes the need to cultivate a caring and healing environment [14]. In such settings, empathetic and responsive care deepens patient trust in the healthcare team, improves adherence to treatment plans, and enhances health outcomes. This mutual trust not only benefits patients but also increases nurses’ sense of accomplishment and meaning in their work [46].

Moreover, HHCP enhances PPB by fostering greater work engagement. When nurses feel connected to patients and perceive their care as meaningful, they are more likely to invest effort in individualized care and proactive clinical practice. Higher engagement not only promotes better patient outcomes but also strengthens nurses’ internal sense of professional growth and contribution [47].

In addition to this core caring‐driven pathway, several contextual factors—employment type, nurse position, and exercise habits—shape the conditions under which professional benefits are experienced. These factors do not replace the primary mechanism but may influence how strongly nurses internalize the emotional and cognitive gains associated with caring practice. For example, contract nurses often face stronger external incentives for advancement and may invest more effort in demonstrating competence and engaging in caring interactions. Such motivation may amplify the positive emotional experiences described in the Broaden‐and‐Build Theory, facilitating the accumulation of cognitive and behavioral resources that contribute to PPB. Nurses in higher positions, who typically have greater autonomy and participate more extensively in clinical decision‐making, may derive deeper affirmation, meaning, and connection—key components emphasized in Watson’s theory—thereby reinforcing their perception of professional value. Regular exercise may also support the development of PPB by enhancing psychological resilience, enabling nurses to more effectively absorb positive emotions from caring interactions and convert them into enduring personal resources.

### 4.5. Strengths and Limitations

This study offers several important contributions to the nursing management literature. First, data were gathered from 276 nurses across 23 hospitals situated in six provinces in China, providing a sample that is both demographically and institutionally diverse. Such a broad sampling frame enhances the external validity of the findings by capturing regional differences in nursing experiences and practices. Second, the study utilized psychometrically sound instruments, including the HHCP Scale and the Perceived Professional Benefit Questionnaire, both of which demonstrated high internal consistency. This approach ensures the reliability and precision of the data collected. Furthermore, the application of advanced analytical techniques, such as Pearson’s correlation analysis and multiple linear regression, enabled the identification of key predictors with statistical rigor. These methodological strengths support the credibility of the conclusions and offer actionable insights for nursing leaders, particularly in designing targeted interventions to enhance humanistic care and strengthen professional development pathways.

Despite these strengths, several limitations should be acknowledged. The cross‐sectional nature of the study precludes any inference about causation, limiting conclusions to associations rather than directional relationships. Additionally, self‐report measures may be influenced by recall errors or the desire to respond in socially acceptable ways, potentially affecting data accuracy. The findings may also have limited generalizability beyond the sampled regions or time frame, as variations in institutional policies and regional healthcare systems were not fully accounted for. Moreover, important contextual variables—such as organizational climate, workload, and leadership style—were not included, which may have confounded the observed relationships. To build on these findings, future research should consider employing longitudinal or experimental designs and incorporating broader organizational and psychological variables to provide a more comprehensive understanding of the factors shaping nurses’ professional benefit.

## 5. Conclusions

This study demonstrates a strong positive association between nurses’ HHCP and their PPB, with HHCP emerging as the dominant predictor and the final regression model accounting for 58.2% of the variance. Nurses with higher HHCP, reflected in supportive interactions with colleagues, leadership, and patients, reported greater self‐growth, stronger team belonging, and a clearer professional identity. In addition, employment type was significantly associated with PPB, with contract‐based nurses reporting higher PPB scores than permanent or personnel‐agency nurses. Nurse position and regular exercise were positively associated with PPB, suggesting that higher‐level roles and better physical and mental well‐being facilitate the realization of professional benefits. These findings highlight the importance of fostering holistic human caring and supportive workplace conditions to enhance nurses’ professional experiences and satisfaction.

## 6. Implications for Nursing Managers and Educators

The findings of this study have several implications for nursing managers and educators. First, HHCP was the strongest predictor of PPB, underscoring the importance of fostering humanistic care in both education and clinical practice. Managers and educators should implement immersive training, scenario‐based exercises, structured mentoring, and reflective practice to cultivate empathy, communication skills, and emotional intelligence among nurses.

Second, nurse position and employment type were associated with PPB, highlighting the need for tailored professional support. Nurses in managerial or supervisory roles benefit from greater autonomy and participation in decision‐making, which enhances professional identity and satisfaction. Clear career pathways, leadership or project opportunities, and role diversification can support their growth. Contract‐based nurses reported higher perceived benefits, likely reflecting their younger age, strong motivation, and adaptability to new technologies. Nursing managers should recognize the diverse needs of nurses across employment types, providing targeted interventions such as flexible scheduling, skill development, and workload adjustments to sustain engagement and fulfillment for all staff.

Third, exercise habits were positively linked to PPB, suggesting that physical activity supports resilience, stress management, and positive work engagement. Institutions can promote health behaviors through access to fitness facilities, wellness programs, or structured group activities, enhancing nurses’ well‐being and professional satisfaction.

Finally, broader supportive infrastructures and strategies are essential. Access to mental health services, formal peer recognition, flexible scheduling, and public recognition of the nursing profession can strengthen team cohesion, reduce emotional fatigue, and reinforce nurses’ sense of professional value. Data‐driven evaluation frameworks are recommended to monitor intervention outcomes and guide continuous improvement. Collectively, these multilevel strategies can foster a resilient, highly engaged nursing workforce capable of delivering person‐centered care in complex healthcare settings.

## Author Contributions

Jiayi Chen, Jiachun Lu, and Yanli Hu conceived the study and developed the initial research design. Jiayi Chen, Jiachun Lu, Xiaohan Hu, Lingjuan He, and Yingai Cui conducted data collection, data management, and preliminary analyses. Jiayi Chen and Jiachun Lu drafted the original manuscript. Yanli Hu provided critical revisions and overall supervision during the initial development of the work. Ying Liu contributed to further data analysis and interpretation, and Peng Xiong assisted in statistical verification to enhance the robustness of the results.

## Funding

This work was supported by the Project for Improving Scientific Research Capabilities of Guangzhou Medical University in 2024 (No. 2024SRP043).

## Disclosure

The funder had no role in the design and conduct of the study; the collection, management, analysis, and interpretation of the data; the preparation, review, or approval of the manuscript; or the decision to submit the manuscript for publication. All authors reviewed and approved the final version of the manuscript for submission.

## Ethics Statement

All study procedures were approved by the Ethics Committee of Guangzhou Medical University (No. L202306002), Guangzhou, China. All participants were informed of the purpose of this study and provided signed informed consent.

## Conflicts of Interest

The authors declare no conflicts of interest.

## Supporting Information

Additional supporting information can be found online in the Supporting Information section.

## Supporting information


**Supporting Information 1** Appendix 1. STROBE checklist. This supplementary material contains the completed Strengthening the Reporting of Observational Studies in Epidemiology (STROBE) checklist for this cross‐sectional study.

## Data Availability

The data supporting the findings of this study are available from the corresponding author upon request.
