# Supplementary material for: Holistic Human Caring Perception and Perceived Professional Benefit Among Nurses in China: A Multihospital Cross‐Sectional Study
Source: J Nurs Manag. 2026 Jul 11;2026:3967540. doi: 10.1155/jonm/3967540 (PMC13355309; doi:10.1155/jonm/3967540)
Supplement: Supplementary file 1 — Supporting Information 1 Appendix 1. STROBE checklist. This supplementary material contains the completed Strengthening the Reporting of Observational Studies in Epidemiology (STROBE) checklist for this cross‐sectional study. [file JONM-2026-3967540-s001.docx]

STROBE Statement—Checklist of items that should be included in reports of ***cross-sectional studies***

|  | **No** | **Recommendation** | **Study-specific details** |
| --- | --- | --- | --- |
| **Title and abstract** | 1 | (*a*) Indicate the study’s design with a commonly used term in the title or the abstract | Title: “Holistic Human Caring Perception and Perceived Professional Benefit Among Nurses in China: A Multi-Hospital Cross-Sectional Study” clearly indicates cross-sectional design. |
|  |  | (*b*) Provide in the abstract an informative and balanced summary of what was done and what was found | Abstract summarizes aim, background , methods (Chinese nurses, multi-center, questionnaires, regression analysis), key results (HHCP positively associated with PPB, employment type, nurse position, exercise habit), conclusions and implication for nursing manager. |
| **Introduction** | | |  |
| Background/rationale | 2 | Explain the scientific background and rationale for the investigation being reported | Nursing workforce experiences high stress; understanding factors influencing perceived professional benefit can inform workforce management. HHCP is hypothesized to influence PPB. |
| Objectives | 3 | State specific objectives, including any prespecified hypotheses | To examine the relationship between holistic human caring perception and perceived professional benefit among Chinese nurses; to explore the influence of demographic, occupational, and lifestyle factors. |
| **Methods** | | |  |
| Study design | 4 | Present key elements of study design early in the paper | Multi-center cross-sectional survey conducted in hospitals in China. |
| Setting | 5 | Describe the setting, locations, and relevant dates, including periods of recruitment, exposure, follow-up, and data collection | 23 hospitals in China; data collected between January and February 2025 |
| Participants | 6 | (*a*) Give the eligibility criteria, and the sources and methods of selection of participants | Eligibility criteria for inclusion required that participants: (1) held active registration as clinical nurses; (2) had completed standardized orientation or onboarding training. Individuals were excluded if they: (1) were temporary visiting professionals from other institutions; (2) were not present at work during the data collection period. |
| Variables | 7 | Clearly define all outcomes, exposures, predictors, potential confounders, and effect modifiers. Give diagnostic criteria, if applicable | Outcome: PPB (perceived professional benefit). Predictor: HHCP (holistic human caring perception).  Contextual factors:employment type, nurse position, exercise habit, |
| Data sources/ measurement | 8* | For each variable of interest, give sources of data and details of methods of assessment (measurement). Describe comparability of assessment methods if there is more than one group | Self-administered questionnaires including validated HHCP and PPB scales; demographic information collected via structured forms. Same methods applied across hospitals. |
| Bias | 9 | Describe any efforts to address potential sources of bias | Use of validated scales, anonymous survey to reduce social desirability bias; multi-center design to reduce selection bias. |
| Study size | 10 | Explain how the study size was arrived at | The minimum required sample size was calculated using G*Power 3.1 for multiple linear regression. A medium effect size (f² = 0.15), a power of 0.80, and an alpha level of 0.05 were assumed. With 27 predictors, the recommended minimum sample size was 196. The final sample of 276 participants exceeded this requirement, providing sufficient power for the planned analyses. |
| Quantitative variables | 11 | Explain how quantitative variables were handled in the analyses. If applicable, describe which groupings were chosen and why | Continuous variables (HHCP, PPB scores) analyzed directly. |
| Statistical methods | 12 | (*a*) Describe all statistical methods, including those used to control for confounding | Descriptive statistics for demographics; Pearson correlations; multiple linear regression for associations with PPB controlling for covariates. |
|  |  | (*b*) Describe any methods used to examine subgroups and interactions | subgroup analyses for nurse position and exercise habit |
|  |  | (*c*) Explain how missing data were addressed | missing data <5%, handled with complete case analysis. |
|  |  | (*d*) If applicable, describe analytical methods taking account of sampling strategy | No complex sampling adjustments needed. |
|  |  | (*e*) Describe any sensitivity analyses | Sensitivity analyses conducted by including/excluding personnel-agency nurses. |
| **Results** | | |  |
| Participants | 13* | (a) Report numbers of individuals at each stage of study—eg numbers potentially eligible, examined for eligibility, confirmed eligible, included in the study, completing follow-up, and analysed | A convenience sampling approach was used. A total of 318 questionnaires were distributed, and 276 valid responses were included in the final analysis (response rate: 86.8%). |
|  |  | (b) Give reasons for non-participation at each stage | Questionnaires were excluded if (1) more than 80% of the items were answered with the same option, suggesting inattentive responding, or (2) the total completion time was less than 5 minutes, indicating insufficient engagement with the survey content. |
|  |  | (c) Consider use of a flow diagram | The participant flow is described in detail in the text (Section 2.1), including numbers distributed, excluded, and analysed. |
| Descriptive data | 14* | (a) Give characteristics of study participants (eg demographic, clinical, social) and information on exposures and potential confounders | Hospital level, age, education background, working years, marital status, number of children, night-shift frequency, employment type, professional title, nurse position, place of birth, only-child status, religious belief, exercise habits, and monthly income, HHCP and PPB scores. |
|  |  | (b) Indicate number of participants with missing data for each variable of interest | Not applicable. |
| Outcome data | 15* | Report numbers of outcome events or summary measures | Mean (SD) PPB score overall and by subgroups; range reported. |
| Main results | 16 | (*a*) Give unadjusted estimates and, if applicable, confounder-adjusted estimates and their precision (eg, 95% confidence interval). Make clear which confounders were adjusted for and why they were included | Univariate analysis and multiple regression results provided. |
|  |  | (*b*) Report category boundaries when continuous variables were categorized | Not applicable. |
|  |  | (*c*) If relevant, consider translating estimates of relative risk into absolute risk for a meaningful time period | Not applicable. |
| Other analyses | 17 | Report other analyses done—eg analyses of subgroups and interactions, and sensitivity analyses | No significant interaction found. Sensitivity analyses with alternative variable coding consistent with main results. |
| **Discussion** | | |  |
| Key results | 18 | Summarise key results with reference to study objectives | HHCP positively associated with PPB; employment type, nurse position, exercise habit shape how benefits are realized. |
| Limitations | 19 | Discuss limitations of the study, taking into account sources of potential bias or imprecision. Discuss both direction and magnitude of any potential bias | Cross-sectional design prevents causal inference; convenience sampling limits generalizability; self-report measures may introduce reporting bias. |
| Interpretation | 20 | Give a cautious overall interpretation of results considering objectives, limitations, multiplicity of analyses, results from similar studies, and other relevant evidence | Findings support theoretical frameworks (Watson’s Human Caring Theory, Broaden-and-Build Theory); contextual factors moderate effect; implications for nurse management and workplace interventions discussed. |
| Generalisability | 21 | Discuss the generalisability (external validity) of the study results | Findings relevant to hospital nurses in similar Chinese settings; may not generalize to other countries or healthcare systems. |
| **Other information** | | |  |
| Funding | 22 | Give the source of funding and the role of the funders for the present study and, if applicable, for the original study on which the present article is based | This work was supported by The Project for Improving Scientific Research Capabilities of Guangzhou Medical University in 2024（No.2024SRP043）and Dongguan Sci-tech Commissioner Program (No.20231800500372).  ; funders had no role in study design, data collection, analysis, or manuscript preparation. |

*Give information separately for exposed and unexposed groups.
